# Microbial identification of potato taste defect from coffee beans

**DOI:** 10.1002/fsn3.887

**Published:** 2018-11-20

**Authors:** Jean Bernard Ndayambaje, Antoine Nsabimana, Sylvie Dushime, Florence Ishimwe, Habinshuti Janvier, Martin P. Ongol

**Affiliations:** ^1^ Department of Chemistry School of Science College of Science and Technology University of Rwanda Kigali Rwanda

**Keywords:** 16S rDNA, coffee, microbes, potato taste defect

## Abstract

Coffee is a socioeconomic important plant all over the world due to its exportation and how it provides income to the farmers and the country. However, potato taste defect (PTD) affects the Rwandan coffee quality. The smell is reported to be caused by some bacteria that are responsible for the off‐flavor and may also be related to the infestation of Antestia pest which are in its elimination process. The aim of this study was to isolate, biochemically characterize, and identify bacteria producing potato flavor from Rwandan coffee. Five samples were obtained from different regions (*Nyamasheke* and *Nyakizu*) of Rwanda. Bacteria were isolated and enumerated in the nutrient agar media followed by culture on nutrient and tryptic soy broth media. Bacteria were also cultured to several carbon sources such as glucose, fructose, sucrose, starch, pectin, and galactose to smell the odor produced by those bacteria. DNA extraction of isolates was done, and the resulting DNA strands were undergone three steps of PCR to be amplified using the forward primer and reverse primer. The identification of bacteria producing potato flavor from Rwandan coffee beans was done through 16S rDNA method followed by sequence analysis using FinchTV software and BLAST. Earthy odor was the mostly produced one for nutrient agar and tryptic soy agar media, and for carbon sources such as sucrose, glucose, pectin, fructose, and galactose. The potato odor was recorded mostly from damaged floaters and hand‐sorted damaged coffee beans. However, other odors such as fruity and ferment were found to be produced by bacteria in coffee beans. The study came up by concluding the presence of different kinds of bacteria including *Enterobacteriaceae* and *Pantoea,* which are responsible for the formation of 2‐isopropyl‐3‐methoxypyrazine (IMP) in coffee beans and cause the production of potato flavor.

## 1 INTRODUCTION

The coffee tree belongs to the Rubiaceae family, genus Coffea. However, more than 80 coffee species have been identified worldwide. Only two varieties are economically important namely *Coffea arabica* and *Coffea canephora* or Robusta coffee (commercial name of one of the main *Coffea canephora* cultivars). *Coffea arabica*, also known as Arabica coffee, is responsible for approximately 70% of the global coffee market, and *Coffea canephora* (Robusta) accounts for the rest (Fărcaş et al., 2014). In Rwanda, the most commonly cultivated coffee species is *Coffea arabica,* mainly the bourbon type of coffee, because it is grown on fertile volcanic soils on high‐altitude hills (Boudreaux, 2007; Loveridge, Mpyisi, & Weber, 2002) Most *Arabica* coffee is processed in one of two ways: dry processing and wet processing. Dry processing is simpler, more often used in East Africa, and involves simply allowing the harvested fruits to dry in the sun intact. When they are dry, the beans are removed by a machine (Daniels, 2009). Wet processing is more common in Latin America and involves more steps. The beans are removed from the fruit, allowed to ferment to remove a slippery mucilage layer (fruit layer), washed, and dried (Fukushima et al., 2009). The popularity of coffee is due to its unique flavor components and health benefits associated with its consumption. The most important components of coffee contributing to its health effects are caffeine and polyphenols (Mohr, Nielsen, & Bangsbo, 2011). The former has effects such as alleviation of stress and fatigue and also plays a role of stimulating central nervous system by accumulating alertness, reducing sleep, increasing short‐term memory, and rising the effectiveness of certain drugs (Reilly, Drust, & Clarke, 2008), and the latter has effects that reduce risks such as diabetes, liver cirrhosis, colorectal cancer, and death (Mohr et al., 2011). Coffee plays important economic and social role in our country. According to National Rwanda Strategy (2009–2012), coffee exports accounted for about US$ 58 million and 60% of total exports in the early 90s but was severe crisis experienced so that revenues were down to 20 million US$ in 2001 and represented 30% of total exports. Coffee has again become one of the country's principal exports, with growing revenue at an average of 30% per year during the period of 2002–2006. Coffee farmers represent only 10% of the 90% of Rwandans who work in agriculture, but the coffee that they produce makes up an extensive portion of the country's exports (Petit, 2007). Quality is the most important parameter in the world coffee trade (Davids, 2003). In order to improve the quality and commercial value of coffee exported from East Africa, the governments are encouraging the production of fully washed coffee as a means to increase coffee quality (Davids, 2003).

However, potato taste defect (PTD) has been mentioned by coffee buyers as a significant problem affecting East Africa coffee quality (Davids, 2003). The PTD smell is also observed in green coffee and in coffee during processing. The smell is reported to be caused by some bacteria of the genus Pantoea (Gueule et al., 2015), which develop in coffee beans and produce the chemical compound called 2‐isopropyl‐3‐methoxypyrazine (IMP) that is responsible for the PTD (Sunarharum, Williams, & Smyth, 2014). Among members of the family *Enterobacteriaceae*, most strains of *Serratia odorifera* and *Serratia ficaria*, and a few strains of *Serratia rubidaea* (synonym: *Serratia marinorubra*) and *Cedecea davisae* are also known to produce potato‐like odors when grown on agar media (Clay, Bro, Church, Ortega, & Bizoza, 2018; Nzeyimana, Hartemink, & Geissen, 2014). The randomness presence of the potato taste in coffee brings attention to do more research to define the origin of PTD and its manifestation.

Rwandan coffee produced is mainly exported to specialty coffee markets that are highly competitive and demand high‐quality coffee especially in terms of sensory attributes. However, PTD has become a serious concern as it can affect the Rwandan coffee quality if it is not well managed and controlled. Coffee buyers state that the perceived random appearance of the PTD diminishes the quality of most African coffee and confidence in buying coffee from this country, which affects the lives of farmers and the economy of the country (Pandey et al., 2000). The PTD is now recognized as serious by the government and a campaign is underway. In the situation of Rwanda, the researchers from National Agricultural Export Board (NAEB) with other research institutions are working on eradicating Antestia pest. According to the NAEB, the Antestia insect sucks the fruit juice itself, which already affects up to 38% of its yield. To remedy this problem, much more research is needed on the microorganisms responsible for PTD and on the exact factors leading to PTD in coffee. A number of different carbon sources were used for culturing the coffee samples. The main purpose was the production of building blocks and energy for the cell itself. In the product of evolution, different species have different metabolic pathways, and different species take up different carbon sources with many different mechanisms.

Therefore, the focus of this study is on the isolation and identification of bacteria producing potato flavor from coffee beans. The accurate bacterial isolation is done through the use of 16S rDNA sequencing.

## 2 MATERIALS AND METHODS

### 2.1 Sample collection

**Figure 1:**
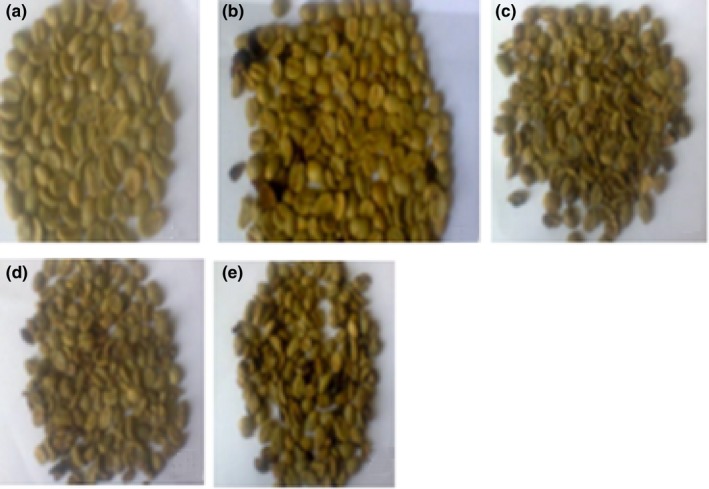
Samples collected from two districts. (a) Undamaged washed coffee beans; (b) Undamaged sun‐dried coffee beans; (c) Damaged washed coffee beans; (d) Damaged floaters (floated during fermentation of coffee beans); (e) Hand‐sorted damaged coffee beans

The samples of all damaged floaters (floated during fermentation of coffee beans), washed coffee beans, and hand‐sorted coffee beans together with undamaged washed and sun‐dried coffee beans were collected randomly from different areas, *Nyamasheke* and *Nyakizu* districts, in Rwanda, as mentioned in Figure 1. The source of invasions was not identified whether it is due to Antestia or bacterial infestation. The above five mentioned samples were brought to UR‐CST laboratory and kept in airtight Z‐block bag to avoid moisture and contamination and put in the fridge at temperature of 4°C for preservation.

### 2.2 Isolation and screening of bacteria

From each sample, 20 g of dry coffee beans was weighed and grinded. One gram of the grinded sample was put in a factory‐sterilized falcon tube containing 9 ml of saline solution (0.85% NaCl) and mixed thoroughly. From the homogenized mixture obtained, sixfold decimal serial dilution was done in six sterile Eppendorf tubes containing 900 μl of saline solution. Each sample (100 μl) of five Eppendorf tubes was inoculated and spread on plates containing nutrient agar media (Laboratorios Conda, S.A) and tryptic soy agar media. Inoculated plates were incubated at 25°C for 72 hr in the incubator.

Bacteria were counted using colony counter (Stuart, Model No. R000102878, UK) and identified based on morphological characteristics (elevation and shape) and appearance (color) of the colonies formed. A single bacterial colony was picked using a sterile wire loop and streaked on fresh nutrient agar and tryptic soy agar media, followed by incubation of 72 hr. This procedure was repeated three times in order to purify the colonies. The pure colonies were subjected to the extraction and reduction of result interferences. The pure colony obtained was picked using a sterile wire loop and streaked in a fresh nutrient broth and tryptic broth media followed by the incubation for 4 days. The identification of odor produced by the bacteria was done using food sensory analysis methods including triangle test, two‐out‐of‐five test, duo‐trio test, paired comparison, simple difference test, a‐not‐a test, sequential tests, similarity testing.

### 2.3 DNA extraction, purification, and PCR amplification

Bacterial DNA was extracted using a commercial kit (DNeasy Blood and Tissue Kit, Qiagen, the Netherlands). Fifty microliters of mutanolysin (Sigma‐Aldrich, Germany) and 50 μl of lysozyme (Sigma‐Aldrich, Germany) for breaking bonds in the peptidoglycan layer, and 200 μl of cell suspension and 20 μl of proteinase‐K for protein degradation (Qiagen GmbH, 1017738, the Netherlands) were added in 35 Eppendorf tubes and were mixed with a vortex then incubated in water bath at 37°C for 2 hr to enhance lysozyme activity followed by incubation in water bath at 56°C for 10 min to activate proteinase‐K and mixed using the vortex. This process was for cell lysis. Two hundred microliters of ethanol (≥99.8%, Sigma‐Aldrich, Germany) for DNA precipitation was added in each Eppendorf tube and mixed with a vortex. The mixture was transferred to the spin column, placed in collection tube, and centrifuged at 8,000 *g* for 1 min at room temperature, and the spin column was transferred into the new collection tube. Others steps were done according to the manufacturer's instructions. The extracted DNA was put in the freeze dryer to freeze‐drying in order to increase the concentration of DNA and to obtain the dried DNA extract. The DNA purification was done by using QIAquick PCR Purification Kit (Qiagen Group, 2011, the Netherlands). The purified and amplified DNA by PCR was kept in the freezer at −85°C. The amplification cycle was done as follows: hot start at 95°C for 5 min; denaturation at 94°C for 0.5 min; annealing at 55°C for 0.5 min; extension at 72°C for 2 min; and final extension at 72°C for 10 min. The following forward primer (5′‐AGAGTTTGATCCTGGCTCAG‐3′) and reverse primer (5′‐GGTTACCTTGTTACGACTT‐3′) were used, and the PCR amplification was completed after performing 30 cycles.

### 2.4 DNA sequencing and sequence analysis

The purified and dried DNA samples were sent to inqaba biotec laboratory (South Africa, Pretoria) for DNA sequencing using 16S rDNA method. Homology searches were performed in GenBank database using the basic local alignment search tool (BLAST; http/blast.ncbi.nih.gov/blast.cgi) program to identify the bacterial strains closely related to the isolates and the best matching DNA sequences.

## 3 RESULTS AND DISCUSSIONS

**Figure 2:**
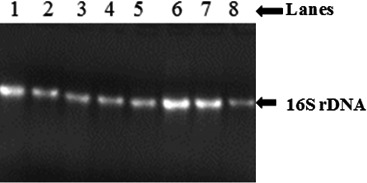
PCR products of 16S rDNA gene

The results obtained after first culturing on nutrient agar and tryptic soy agar (TSA) plates at 25°C for 48–72 hr showed the growth of bacteria in the coffee beans proportional to the concentration. On the NA, some of the colonies were yellow and others whitish, and in circular and irregular shape with flat and convex elevation, whereas the colonies on the TSA were yellowish, whitish, orange, and pink color, and in circular, irregular, and filamentous shape with flat, raised, and convex elevation. The identification of bacteria was confirmed by PCR amplification at 55°C for 0.5 min using the universal 16S rDNA primers, and the mixture was resolved in 1.5% agarose gel (Sigma‐Aldrich, Denmark) for size confirmation. The results showed the intense bands of 16S rDNA gene, and the lanes 1–8 represent 16S rDNA genes from different colonies from different samples (Figure 2).

According to the odor produced by *Enterobacteriaceae* and *Pantoea bacteria*, earthy odor (odor produced by soil after the first rain) and potato odor (odor of peeled potatoes) were obtained from all media used (nutrient broth and tryptic soy broth). Potato odor was found mostly in samples (damaged floaters or coffee beans floated during fermentation) and (hand‐sorted damaged coffee beans), and those samples were damaged coffee beans. The presence of the potato odor was caused by bacteria (*Enterobacteriaceae* and *Pantoea),* which developed in coffee beans and formed IPMP responsible for potato odor. Those bacteria might have attacked the coffee beans through damaged coffee beans by insects. The presence of potato flavor might be caused by bacteria because coffee beans floated during fermentation contain more microbes than sun‐dried coffee beans. According to Table 1, some other bacteria were present without producing any odor. It was found that 2‐isopropyl‐3‐methoxypyrazine (IMP) and 3‐isobutyl‐2‐methoxypyrazine (IBMP) were responsible for the earthy and potato flavors in wines, grapes, and mustard plants. They also play an important role in the aroma produced by coffee beans as chromatograms of nonpeasy and peasy Rwandan coffee samples showed similar concentrations of other pyrazines such as IBMP. Hence, other pyrazines (except IMP) were ruled out as possible cause of potato defect in coffee (Czerny & Grosch, 2000). It has been reported that potato‐like flavor results from bacteria of *Enterobacteriaceae* and *Pantoea* family (Grimont, Grimont, & Starr, 1981). Most of their strains produced potato flavor when they cultured on agar media and developed in coffee beans, they produced the chemical compound called 2‐isopropyl‐3‐methoxypyrazine (IMP) (Waikar, 2012).

**Table 1:** Result on DNA analysis after DNA sequencing by 16S rDNA method

Code	Closest	Identity%	Accession number	Isolation medium	Sample description (colonies)
11F NA N	*Pantoea* sp. LL56	90	KF202778.1	NA	No5, Nyakizu
11 R	*Enterobacteriaceae* bacterium; X1/SB91	99	LC007926.1	NA	No5, Nyakizu
8R	*Enterobacter cloacae* strain 1C‐40	100	KR061400.1 N	NA	No5, Nyakizu
4F	*Pseudomonas* sp.c156	100	FJ950610.1	NA	No4, Nyamasheke
7R	*Kosakonia cowanii* strain N2	99	KM349407.1	NA	No5, Nyakizu
9F	*Enterobacter cancerogenus*	99	HF562891.1	NA	No1, Nyamasheke
3F	*Escherichia* sp. PHFF‐9	99	KM817774.1	NA	No4, Nyamasheke
8F	*Enterobacter* sp. WS05	97	JN210900.1	NA	No5, Nyakizu
9R	*Bacterium* CSR‐MOB8	100	KJ018077.1	NA	No1, Nyamasheke
10F	*Klebsiella pneumoniae*	100	HE578781.1	NA	No5, Nyakizu
10R	*Klebsiella variicola* strain A6128	99	KM275666.1	NA	No5, Nyakizu

Note

NA: nutrient agar.

The latter compound is responsible for the off‐flavor, and the potato flavor obtained was due to *Enterobacteriaceae* and *Pantoea* as it was identified after sequence analysis (Table 1).

Potato flavor also may be detected during processes such as harvesting, transport from harvesting to washing stations, pulping, and fermentation (Hughes et al., 2014). In this study, earthy odor was found in all samples (No1 to No5) resulting from soil contamination associated with bacteria. Nutrient broth was the more favorable medium for bacteria to produce potato odor compared to tryptic soy broth, and the same samples (No4 and No5) produced potato odor for both broth media. There were other kinds of odors from all samples such as ferment and fruity, and it showed that coffee can have microbes producing various odors. The fruity odor might be due to the formation of ester derived from alcohol and carboxylic acid reaction, and the ferment odor might be due to the fermentation of sugars by anaerobic bacteria to produce alcohol that is oxidized and changed into acids.

Bacteria isolated from many kinds of carbon sources such as glucose, fructose, sucrose, starch, pectin, and galactose were incubated at 37°C for 24 hr. There was variation of pH as some bacteria were grown on more acidic and others on less acidic conditions because some carbon sources are monosaccharides (glucose, fructose, and galactose) and disaccharide (sucrose), and others are polysaccharides (pectin and starch). Those simple carbon sources were easily digested by the bacteria compared to complex carbon sources because simple carbon sources on growing bacteria showed low pH compared to complex carbon sources. Therefore, the bacteria producing potato flavor can grow on acidic media and can be inhibited on basic media as way of controlling their growth. The result obtained from South Africa after DNA sequencing by 16S rDNA for different samples shows the presence of various bacteria in different samples of coffee beans that might cause the earthy odor in them (Table 1)**,** whereas in sample No5 (Nyakizu hand‐sorted damaged coffee beans), the presence of *Enterobacteriaceae* and *Pantoea* bacteria which develop in coffee beans and lead to formation of IMP in coffee responsible for the potato flavor was identified after the sequence analysis using BLAST. Therefore, we confirmed that the production of potato odor in coffee beans was due to the *Enterobacteriaceae* and *Pantoea* bacteria as shown in Table 1. It has been reported that the acidity may increase as levels of aliphatic acids (formic, acetic, glycolic, and lactic) rising through degradation of sucrose, polysaccharides, and other compounds. The production of bubbles by grown bacteria on the surface of carbon sources showed that there was gas production and the fermentation occurred by producing alcohol that is oxidized and changed into acids. These carbon sources were used because they are among major components of coffee beans and it plays a crucial role for the coffee flavors. According to the production of odor, for all carbon sources earthy was the most produced odor resulting from soil contamination associated with causal bacteria.

Potato odor was produced from pectin, galactose, fructose, and starch that were due to the causal bacteria of *Enterobacteriaceae* and *Pantoea,* which developed into the coffee beans and led to the formation of IPMP responsible for potato odor.

## 4 CONCLUSION AND RECOMMENDATION

### 4.1 Conclusions

In short, using both nutrient agar and tryptic soy agar media was suitable to isolate microorganisms (bacteria). According to odor produced by bacteria from different media and carbon sources, nutrient broth and tryptic soy broth were becoming good media to smell the odor produced, but samples No4 to No5 cultured on nutrient broth, pectin, galactose, and starch, produced more potato odor compared to tryptic soy broth, sucrose, fructose, and glucose. Earthy odor was the frequently produced odor (samples No1 to No 5) for all media and carbon sources. The results obtained after DNA extraction from samples No4 and No5 indicated that there was optimization of PCR amplification for those bacteria because the DNA bands were obtained (Figure 2). The study came up to confirm the presence of bacteria producing potato flavor from Rwandan coffee beans through 16S rDNA sequencing method done in inqaba biotec laboratory (South Africa, Pretoria) and sequence analysis by FinchTV software and BLAST. The major findings from ours studies found that *Enterobacteriaceae* and *Pantoea* bacteria are mainly associated with potato flavor in Rwandan coffee beans.

### 4.2 Recommendation

This study opened wide avenues for further research in this field of potato taste defect in coffee by analyzing more potato defect infected beans from field and try to find out more metabolites The statistical survey can be completed to evaluate the occurrence frequency of potato defect in different coffee farms and coffee washing stations in Rwanda to establish the major source of production of infected coffee cherries or beans. Finally, further research on other odors, such as fruity and ferment, is required to identify the contribution of these odors in improving the coffee flavor. The samples used during this study were obtained from few sites, and it is recommended to use samples from more different regions to confirm the presence and variations of bacteria producing potato flavor from Rwandan coffee beans.

## CONFLICT OF INTEREST

Authors declare no conflict of interest.

## ETHICAL STATEMENT

This work does not involve any human or animal studies.
